# Impact of oral hygiene habits on oral health-related quality of life of in-school adolescents in Ibadan, Nigeria

**DOI:** 10.3389/froh.2022.979674

**Published:** 2022-09-09

**Authors:** Folake Barakat Lawal, Omotayo Francis Fagbule, Seyi John Akinloye, Taiwo Akeem Lawal, Gbemisola Aderemi Oke

**Affiliations:** ^1^ Department of Periodontology and Community Dentistry, University of Ibadan and University College Hospital, Ibadan, Oyo State, Nigeria; ^2^ Consortium for Advanced Research Training in Africa, African Population and Health Research Center, Nairobi, Kenya; ^3^ Department of Oral Pathology, University College Hospital, Ibadan, Oyo State, Nigeria; ^4^ Division of Pediatric Surgery, Department of Surgery, University of Ibadan and University College Hospital, Ibadan, Oyo State, Nigeria

**Keywords:** adolescents, oral health, oral hygiene, habits, school, quality of life

## Abstract

**Background:**

Recent evidence has shown that the prevalence of periodontal diseases is still high among adolescents and, thus, there is an impetus to promote good oral hygiene habits among them through schools. There is a need to provide baseline data on the oral hygiene habits of adolescents and how it impacts their oral health-related quality of life for appropriate intervention to be instituted. Moreover, oral health-related quality of life describes how oral health affects the daily activities of individuals; thus, it provides a holistic means of involving individuals in making decisions about their oral health including uptake of oral hygiene habits.

**Aim:**

To assess the impact of oral hygiene habits on adolescents' oral health-related quality of life.

**Methods:**

A cross-sectional study was conducted among 1,800 adolescents aged 14–18 years attending 36 Senior Secondary Schools in the metropolis of Ibadan, Nigeria. Data were collected using a self-administered questionnaire, which assessed students' sociodemographic characteristics, oral hygiene habits, and oral health-related quality of life with COHIP-SF19. Data obtained was analyzed with SPSS version 25 and the *p*-value was set at <5%.

**Results:**

The mean age of the adolescents was 15.16 (±1.16) years. Many 1,094 (60.3%) cleaned their teeth twice or more often daily with 126 (7.0%) cleaning after meals and 1,519 (84.4%) changing their tooth cleaning agent at three months intervals or less. About 1,215 (67.5%) spent three minutes or longer in cleaning their teeth. Only a few 238 (13.2%) cleaned interdentally and 137 (7.6%) used dental floss. The OHRQoL scores of the adolescents ranged from 9–76. A total of 1,612 (93.5%) had at least an impact on their OHRQoL. Those who cleaned their teeth more frequently (twice or more) were more likely to have better OHRQoL (OR = 1.6, 95% CI = 1.1–2.4, *p* = 0.025) and those who did not clean interdentally were more likely to have better OHRQoL (OR = 2.8, 95% CI = 1.2–6.5, *p* = 0.014) than others.

**Conclusion:**

The oral hygiene habits of the adolescents were suboptimal and those who cleaned their teeth twice or more often each day had fewer impacts on their OHRQoL, whereas those who engaged in interdental cleaning had higher impacts on their OHRQoL than others.

## Introduction

The World Health Organization (WHO) defined health as “a state of complete physical, mental and social well-being and not merely the absence of disease or infirmity” (1). This definition highlights the importance of self-reported health outcomes in determining health status. Self-reported health outcomes can be assessed using Health Quality of Life (HQoL), which describes how the health status affects the quality of life of individuals (2) or patients reported outcomes (PROs) specific to patients' report of how health conditions affect their quality of life (3). Similarly, Oral Health-Related Quality of Life (OHRQoL) assesses how oral health impacts the quality of life of individuals with dental patient-reported outcomes (dPROs) being specific to dental patients (3). The PROs were developed for oral health based on the fact that oral health is an integral part of overall health (4, 5). The OHRQoL is thus a predictor of HQoL (6), such that factors that negatively affect OHRQoL will invariably affect HQoL (6, 7). The importance of OHRQoL and dPROs in guiding decisions about the oral health of individuals has been documented (6, 7). They are tools that provide holistic information about how individuals make decisions about their oral health and oral health habits/behavior.

Oral diseases and conditions, including tooth decay, gum diseases, and mouth odor, are associated with having poor OHRQoL (8). The burden of these oral health problems is high globally, especially in Low- and Middle-Income Countries (LMICs) (9, 10). There is a lot of neglect of oral care in LMICs, including in Nigeria, which has made the burden of oral health problems higher than in High-Income Countries (HICs) (9). Most common oral health diseases and conditions are easily preventable by adopting good oral hygiene habits such as twice-daily brushing with the ideal toothbrush, using fluoride-containing toothpaste, and employing the proper brushing technique. Other preventive practices include eating a balanced diet that is low in free sugar content and regular visit to the dental office for checkups coupled with the treatment of diseases in their early stages (11–13).

Although oral practices such as smoking, diet, and utilization of dental services among young adults have been associated with OHRQoL (14), very little is known about how oral hygiene habits influence OHRQoL of adolescents who are in the transition period to adulthood. This becomes important as recent evidence has shown that poor oral health, especially periodontal diseases, exists among adolescents (15), which necessitates the promotion of good oral hygiene among them especially in schools where many of them could be found (16). The school has been advocated as a valuable avenue to further teach children and adolescents about maintaining good oral hygiene through the health-promoting schools initiative (17). While this initiative is yet to be implemented formally in Nigeria (18), there are reports of several informal dental education programs conducted by dental health professionals in Nigerian schools (19–21). It is unknown how effective these programs are, thus there is a need to evaluate the current level of oral hygiene habits among school-going adolescents and the impact of the habits on OHRQoL. Information from this would be useful in reviewing the current educational programs and the Nigerian oral health policies to develop more effective interventions for promoting oral health among adolescents. Furthermore, the constraints militating against formal nationwide school oral health programs abound and justification for changing the trajectory may be strengthened by such evaluation. Hence, this study aimed to investigate oral hygiene habits and their impact on the OHRQoL of school-going adolescents in Ibadan, Nigeria. We hypothesized that good oral hygiene habits are associated with better OHRQoL.

## Materials and methods

### Study design and settings

This cross-sectional study was conducted among adolescents attending randomly selected public secondary schools in Ibadan, Nigeria.

### Participants

A total of 1,800 students participated in the study. They were recruited from schools selected using multistage sampling technique. The first stage involved the random selection of four Local Government Areas within the metropolis of Ibadan using an opaque sealed envelope. An independent research assistant did the selection. The second stage involved the selection of nine schools from each of the Local Government Areas using balloting of sealed opaque envelopes by another independent research assistant. The third stage was the selection of 50 students from the Senior Secondary School I classes in each school using a table of random numbers. Only students who returned signed consent forms and who gave assent to participate in the study were recruited. Students who were ill or were not available at the time of the study were excluded from the study.

### Study size

A sample size of 1,460 was calculated with STATA, using a design effect of 0.78, power of 80%, and a 5% significance level. To allow for a dropout rate of 15%, a minimum of 1,717 students was obtained, and assuming a minimum of 50 students per school, a total of 36 schools was calculated and a sample size of 1800.

### Variables

The outcome variable was the OHRQoL. Exposure/predictors were oral hygiene habits.

### Data sources and measurements

Following ethical approval from the Oyo State Review Board (Ref No: AD 13/479/743), the schools were approached, and the purpose as well as details of the study explained to the principal of each school. With permission from the principals of the schools, the students were approached and gathered either in a large class or school hall depending on their availability in the school. Thereafter, the purpose and details of the study were explained to them, and questions were entertained. This was followed by giving the students consent forms to take home to their parents.

Data for the study was collected using a self-administered questionnaire. The questionnaire (see Supplementary file) consisted of sociodemographic characteristics, which assessed the gender, age, and occupation of the parents of the respondents. The occupation of the parents was further categorized into skilled, unskilled, and dependents based on the modification of the Office of Population Censuses and Surveys (OPCS) that had been used in this environment (15). The questionnaire also assessed oral hygiene habits with questions adapted from the WHO (22) and included; the main type of tooth cleaning aid that the adolescent used, frequency of tooth cleaning, and type of toothbrush with response options of “soft”, “medium”, “hard/very hard and further categorized as medium textured toothbrush and others for the purpose of multivariate analysis using binary logistic regression. Other questions assessed the period/time when the teeth were cleaned; “after meals” or others (before meals or no pattern); duration of tooth cleaning, which was categorized as “three minutes or longer” or “less than three minutes”, interdental cleaning habit was categorized as “Yes”; if it was practiced and “No” if not; interdental cleaning aids used were categorized as “dental floss”, “interdental brush” or others (toothpick, knife, blade, etc.); and dental clinic visits recorded as “Yes” or “No”. Questions on OHRQoL were also included in the questionnaire. The OHRQoL of the adolescents was assessed using the Child Oral Health Impact Profile-Short Form 19 (COHIP-SF 19) (23). The last two questions were positively worded questions while the first 17 questions were negatively worded to describe the impact of oral conditions on the quality of life of the adolescents. Each COHIP-SF 19 question was rated on a 5-point scale in the range: 0 – “never”, 1 – “almost never”, 2 – “sometimes”, 3 – “fairly often”, to 4 – “almost all the time”. Overall OHRQoL score was obtained by reversing the response scores for the 17 negatively worded questions of the COHIP-SF 19. The overall score ranged from 0–76. In this case, higher scores meant a better quality of life. For the purpose of analysis, the COHIP-SF score was recoded into “no impact” for those “never” responses and “impact” for those who chose response options “almost never” to almost all the time”. The questionnaire was validated among 50 students in another school that was not included in the final study.

The questionnaire was administered to the students while seated comfortably on their chairs with the questionnaires placed on the tables in the classrooms to ensure quality was maintained throughout the administration of the questionnaires. A research assistant was always present to guide the students' filling out the questionnaire. The questionnaire was re-administered after one week to 20 students who were selected randomly during the study to assess the test-retest reliability of the questionnaire.

### Bias

The students were selected using a simple random sampling technique to minimize bias that could exist if non-probability sampling techniques were utilized in selecting the students.

### Data management and statistical methods

Data obtained was analyzed using SPSS version 25. Frequencies and proportions were generated for categorical variables and means with standard deviations utilized to summarize numerical variables such as age and COHIP-SF19 scores. The test of association between oral hygiene habits and sociodemographic characteristics of the adolescents was conducted using Chi-square statistics. Also, the association between OHRQoL (COHIP-SF 19 categories of impact and no impact) and oral habit variables was evaluated using Chi-square statistics. Binary logistic regression was used to determine the oral hygiene habits associated with OHRQoL with only variables that were significant at 0.5 or less entered the model. The crude Odds Ratio (OR) and Adjusted Odds Ratio (AOR) were presented. The *p*-value was set at <5%.

## Results

A total of 1, 800 students were approached and all agreed to participate in the study. The Cronbach alpha score of the questionnaire was 0.873 and it ranged from 0.835 to 0.870 when any of the items was deleted.

### Sociodemographic characteristics of the study participants

The mean (SD) age of study participants was 15.16 (±1.16) years. They were all in the tenth grade and 930 (51.7%) were males. Many 1,569 (87.2%) of their parents were unskilled workers, 167 (8.3%) were skilled workers and 64 parents (3.6%) were dependents.

### Oral hygiene habits

Ninety-six percent (1726) of the adolescents cleaned their teeth with a toothbrush: 832 (46.1%) used a medium textured toothbrush, 1,094 (60.3%) cleaned their teeth twice or more frequently daily, 126 (7.0%) cleaned after meals and 1,519 (84.4%) changed their tooth cleaning agent at three months interval or less. A total of 1,215 (67.5%) spent three minutes or longer in cleaning their teeth. Only a few 238 (13.2%) cleaned interdentally and 137 (7.6%) used dental floss. Eighty-two (4.6%) had consulted a dentist in the past (Table 1).

**Table 1:** Oral hygiene habits of the adolescents.

Oral hygiene habits	*n* (%)
Tooth cleaning material	
Toothbrush and paste	1,726 (95.9)
Chewing stick	15 (0.8)
Cotton wool and others	59 (3.3)
Type of toothbrush used	
Soft	394 (21.9)
Medium	830 (46.1)
Very hard/ hard/no toothbrush	576 (31.9)
Frequency of tooth cleaning	
Less than twice daily	706 (39.2)
Twice or more daily	1,094 (60.8)
Period of tooth cleaning	
After meals	126 (7.0)
Before meals	1,674 (93.0)
Change of tooth cleaning material	
≤3 months	1,519 (84.4)
>3 months	281 (15.6)
Duration of tooth cleaning	
≥3 min	1,215 (67.5)
<3 min	585 (32.5)
Interdental cleaning	
Yes	238 (13.2)
No	1,562 (86.8)
Interdental cleaning aid	
Dental floss	137 (7.6)
Broom stick, toothpick, knife, matchstick or none	1,663 (92.4)
Dental visit	
Yes	82 (4.6)
No	1,718 (95.4)

### Association between sociodemographic characteristics and oral hygiene habits

The association between sociodemographic characteristics of the adolescents and oral hygiene habits (Table 2) showed that a higher proportion of female adolescents cleaned their teeth interdentally compared to males (16.0% vs. 10.6%, X^2 ^= 11.137, *p* = 0.001). Males, however, utilized dental services more than females (5.7% vs. 3.3%, X^2 ^=^ ^5.789, *p* = 0.016). A higher proportion of adolescents whose parents were in the skilled occupational class cleaned their teeth more frequently than those with parents categorized as unskilled workers or dependent (65.9% vs. 60.9% vs. 43.8%, respectively, X^2 ^= 9.615, *p* = 0.008). Also, a higher proportion of adolescents whose parents belonged to the skilled occupational class used medium textured toothbrushes compared to those from unskilled or dependent parents (53.3% vs. 46.0% vs. 31.3%, respectively, X^2 ^= 9.171, *p* = 0.010). Adolescents with lower mean age, compared to others, were found to engage more frequently in tooth cleaning (15.1 ± 1.1 vs. 15.3 ± 1.3, *t* = 2.501, *p* = 0.012), cleaned their teeth after meals (15.0 ± 1.1 vs. 15.2 ± 1.12, *t* = 2.062, *p* = 0.039) and spent longer time in teeth cleaning (15.3 ± 1.1 vs. 15.1 ± 1.2, *t* = 2.310, *p* = 0.021).

**Table 2:** Oral hygiene habits and sociodemographic characteristics of the adolescents.

Oral habits	Gender *n* (%)		X^2^	*p*-value	Occupational class *n* (%)			X^2^	*p*-value
	Male	Female			Skilled	Unskilled	Dependent		
Toothbrush type									
Medium	426 (45.8)	404 (46.4)	0.072	0.789	89 (53.3)	721 (46.0)	20 (31.3)	9.171	0.010*
Others	504 (54.2)	466 (53.6)			78 (46.7)	848 (54.0)	44 (68.8)		
Frequency of cleaning									
<2 ce daily	381 (41.0)	325 (37.4)	2.459	0.117	57 (34.1)	613 (29.1)	36 (56.3)	9.615	0.008*
≥2 ce daily	549 (59.0)	545 (62.6)			110 (65.9)	956 (60.9)	28 (43.8)		
Period of tooth cleaning									
Before meals	862 (92.7)	812 (93.3)	0.287	0.592	153 (91.6)	1,460 (93.1)	61 (95.3)	1.023	0.600
After meals	68 (7.3)	58 (6.7)			14 (8.4)	109 (6.9)	3 (4.7)		
Change of cleaning material									
>3 months	144 (15.5)	137 (15.7)	0.024	0.878	23 (13.8)	245 (15.6)	13 (20.3)	1.502	0.472
>≤3 months	786 (85.5)	733 (84.3)			144 (86.2)	1,324 (84.4)	51 (79.7)		
Duration of tooth cleaning									
≥3 min	612 (65.8)	603 (69.3)	2.516	0.113	110 (65.9)	1,069 (68.1)	36 (56.3)	4.181	0.124
<3 min	318 (34.2)	267 (30.7)			57 (34.1)	500 (31.9)	28 (43.8)		
Interdental cleaning									
Yes	99 (10.6)	139 (16.0)	11.137	0.001*	20 (12.0)	210 (13.4)	8 (12.5)	0.291	0.865
No	831 (89.4)	731 (84.0)			147 (88.0)	1,359 (86.6)	56 (87.5)		
Interdental cleaning aid									
Dental floss	61 (6.6)	76 (8.7)	3.028	0.082	14 (8.4)	117 (7.5)	6 (9.4)	0.478	0.788
Others	869 (93.4)	794 (91.3)			155 (91.6)	1,452 (92.5)	58 (90.6)		
Dental services use									
Yes	53 (5.7)	29 (3.3)	5.785	0.016*	8 (4.8)	71 (4.5)	3 (4.7)	0.027	0.987
No	877 (94.3)	841 (96.7)			159 (95.2)	1,498 (95.5)	61 (95.3)		

* Statistically significant; X^2^, Chi-square statistics.

### Oral health-related quality of life

The majority (93.7%) reported an impact of oral health on their quality of life. The OHRQoL scores of the adolescents ranged from 9–76 and the mean score was 61.0 ± 12.0. The most reported item affected in the OHRQoL measure was “pain” noted among 860 (47.8%) followed by “discolored teeth” 743 (41.3%) while the least reported impacted items were “missing school” 302 (18.3%) and “not wanting to speak/read out loud in class because of the teeth/mouth” 348 (19.3%) (Table 3).

**Table 3:** COHIP-SF 19 OHRQoL items impacted upon by oral health.

COHIP-SF 19 Item	*n*	%
Pain in the teeth/toothache	860	47.8
Discolored teeth or spots on the teeth	743	41.3
Crooked teeth or spaces between the teeth	540	30.0
Bad breath	549	30.5
Bleeding gums	640	35.6
Difficulty eating foods you would like to eat	590	32.8
Trouble sleeping	445	24.7
Difficultly saying certain words	484	26.9
Difficulty keeping your teeth clean	519	28.8
Being unhappy or sad	640	35.6
Worries or anxiety	650	36.1
Avoiding smiling or laughing with others	563	31.3
Feeling that you look different	586	32.6
Being worried about what other people think about the teeth/mouth	565	31.4
Being teased, bullied, by other children because of the teeth	405	22.5
Missing school for any reason because of the teeth/mouth	302	18.8
Not wanted to speak/read out loud in class because of the teeth/mouth	348	19.3
Being confident	642	35.7
Feeling of being attractive (good looking).	581	32.3

### Associations between oral hygiene habits and oral health-related quality of life

A higher proportion of those who cleaned their teeth twice or more often each day compared to adolescents who cleaned their teeth less frequently (7.3% vs. 4.7%, X^2 ^= 5.077, *p* = 0.024) reported no impact of oral health on their quality of life (Table 4). On the other hand, a lower proportion of adolescents who cleaned the interdental areas (2.5% vs. 6.9%, X^2 ^= 6.578, *p* = 0.010) reported no impact on their OHRQoL compared to adolescents who did not clean interdentally (Table 4). Other oral hygiene habits did not have significant effect on OHRQoL, although better oral hygiene habits such as the use of a medium textured toothbrush and cleaning the teeth after meals were found to be linked to fewer impacts on their OHRQoL (Table 4).

**Table 4:** Bivariate analysis of impact of oral hygiene habits on OHRQoL.

Oral hygiene habits	COHIP-SF 19 (OHRQoL)		X^2^	*p*-value
	Impact *n* (%)	No impact *n* (%)		
Type of toothbrush used				
Soft	371 (94.2)	23 (5.8)	3.166	0.205
Medium	771 (92.7)	61 (7.3)		
Very hard/ hard/no toothbrush	545 (94.9)	29 (5.1)		
Frequency of tooth cleaning				
Less than twice daily	673 (95.3)	80 (4.7)	5.077	0.024*
Twice or more daily	1,014 (92.7)	33 (7.3)		
Period of tooth cleaning				
After meals	117 (92.9)	9 (7.1)	0.172	0.678
Before meals	1,570 (93.8)	104 (6.2)		
Change of tooth cleaning material				
≤3 months	1,421 (93.5)	98 (6.5)	0.500	0.480
>3 months	266 (94.7)	15 (5.3)		
Duration of tooth cleaning				
≥3 min	1,146 (94.3)	69 (5.7)	2.278	0.131
<3 min	541 (92.5)	44 (7.5)		
Interdental cleaning				
Yes	232 (97.5)	6 (2.5)	6.578	0.010*
No	1,455 (93.1)	107 (6.9)		
Interdental cleaning aid				
Dental floss	129 (94.2)	8 (5.8)	0.048	0.826
Others	1,558 (937)	105 (6.3)		
Utilization of dental services				
Yes	5 (6.1)	77 (93.9)	0.005	0.945
No	108 (6.3)	1,610 (93.7)		

* Statistically significant; X^2^, Chi-square statistics.

Multivariate analysis showed that those who cleaned their teeth more frequently (twice or more) were more likely to have better OHRQoL (OR = 1.61, 95% CI = 1.1–2.4, *p* = 0.025) and those who did not clean interdentally were more likely to have better OHRQoL compared to other adolescents (OR = 2.8, 95% CI = 1.2–6.5, *p* = 0.014) (Table 5).

**Table 5:** Multivariate analysis of oral hygiene habits and OHRQoL.

Oral hygiene habit	OR (95% CI)	*p*-value	AOR (95% CI)	*p*-value
Type of toothbrush used				
Medium	0.7 (0.5–1.6)	0.084	0.7 (0.5–1.1)	0.108
Others	1		1	
Frequency of tooth cleaning				
Twice or more daily	1.6 (1.1–2.4)	0.025*	1.6 (1.0–2.4)	0.032*
Less than twice daily	1		1	
Change of tooth cleaning material				
≤3 months/fraying bristles	1.2 (0.7–2.1)	0.480	1.2 (0.7–2.2)	0.466
>3 months	1		1	
Duration of tooth cleaning				
≥3 min	0.7 (0.5–1.1)	0.132	0.7 (0.5–1.1)	0.109
<3 min	1		1	
Interdental cleaning				
No	2.8 (1.2–6.5)	0.014*	2.0 (1.3–6.9)	0.010*
Yes	1		1	

* Statistically significant; OR, Crude Odds Ratio; AOR, Adjusted Odds Ratio; 95% CI, 95% Confidence Interval.

## Discussion

This study showed that many of the adolescents engaged in frequent tooth cleaning, which many did before meals, and many changed their tooth cleaning aid less than or at three months intervals. In addition, only a few engaged in interdental cleaning and used dental floss while others used harmful aids such as knives; a reflection of poor/suboptimal oral hygiene habits. Adolescents whose parents were in a higher occupational class and those who were younger had better oral hygiene habits than others. Males utilized dental services better than females and, on the other hand, females engaged in interdental tooth cleaning better than males. The majority of the adolescents reported impaired OHRQoL with “toothache” being the most frequently mentioned impairment and “missing school” being the least reported impaired OHRQoL item. Frequent tooth cleaning and interdental tooth cleaning were oral hygiene habits that were significantly associated with OHRQoL. The results of the present research partially support the hypothesis that imbibing good oral hygiene habits increases the chances of having better OHRQoL as this was observed about the frequency of tooth cleaning. However, engaging in interdental tooth cleaning increased the chances of reporting impaired OHRQoL while other oral hygiene habits did not affect the OHRQoL of the adolescents.

It is commendable that the response rate of the adolescents after consenting to participate in the study was high (100%), and this may be an indicator of their enthusiasm for oral health-related activities. While previous studies have looked at the effects of socioeconomic variables, oral health status, and psychological and dental disease conditions on the OHRQoL of adolescents, this is the first study that would assess the influence of oral hygiene habits on their OHRQoL using the COHIP-SF 19 to the best of our knowledge. Also, the study recruited a large sample size, and the selection process for the participants described in this research can be replicated in subsequent studies. Furthermore, the Cronbach alpha score documented in this study is good, higher than the recommended 0.7 (24) thus showing good reliability and a potential of using oral hygiene habits to predict the OHRQoL using the COHIP-SF 19 tool. However, more studies are needed to further validate our findings.

The limitation of this study is that it was conducted among public secondary school students only, thus the findings may not be generalizable to students in private schools. However, the initiation of the school oral health program would more likely commence among adolescents from lower social classes who majorly attend public secondary schools in Nigeria. Despite this limitation, the study has the strength of providing baseline data, with robust sample size, needed to plan interventions.

The adequacy of oral hygiene depends on factors such as brushing duration and frequency, type of toothbrush and toothpaste used, and use of interdental cleaning aids. Many adolescents used toothbrushes and toothpaste as the main tooth-cleaning aid, this is encouraging and conforms with previous reports from the Southwestern part of Nigeria (16, 25). Surprisingly, about three percent of the adolescents either do not have a regular tooth cleaning material or used cotton wool to clean their teeth. Although the use of cotton wool by younger adolescents has been reported in this environment (16), it is worrisome for older adolescents not to have the ideal tooth cleaning material, as this age group is known for being self-conscious (26). It would be important to further investigate the reasons for this. In addition, advocacy for easily available and effective tooth cleaning material should be a focus while promoting good oral hygiene habits among adolescents in schools. More so, a contributing factor to this finding may be because many of the parents of the adolescents in this study were of the low socioeconomic class. Sixty percent of the adolescents cleaned their teeth twice or more often daily. This is encouraging as previous studies from Nigeria reported lower proportions of adolescents doing so; 3.3% were reported in the same city (16), while 31.5% (25) and 8.7% were documented in a semi-urban region in Southwestern Nigeria (27). Furthermore, a lower prevalence of adolescents engaging in teeth cleaning more than once daily has been documented among adolescents in Malta and some Eastern and Southern European countries (28). On the other hand, higher values of 80–89% had been reported in Switzerland (28) and 89.2% noted in Indonesia (29). The differences in the figures reported in the studies can be attributed to the different social classes of the adolescents and the socioeconomic categories of the various countries studied as well as the age groups of the adolescents. Lower social classes of adolescents, the income of countries, and young age groups have been associated with a lower frequency of tooth cleaning (28, 30).

More than half of the adolescents spent adequate time cleaning their teeth, with up to 84.4% of them following the recommendations for changing tooth cleaning devices. This is also encouraging, as a previous study conducted in the city showed a lower proportion (39.2%) (16) than this. This could be attributable to the informal oral health interventions in schools in the city or the age differences in the study population as older adolescents have been observed to have better preventive oral health habits (25).

A low proportion of the respondents cleaned their teeth after meals (7.0%) and 7.6% engaged in interdental cleaning using dental floss as the interdental cleaning aid. The habits may be considered suboptimal and pose risk for inadequate plaque clearance from the mouth that should be achieved if teeth are cleaned after meals and interdentally. A higher proportion of adolescents utilizing dental floss than that observed in this study has been reported among suburban adolescents in Nigeria (25, 27), and in France (31). An explanation for this low proportion engaging in interdental cleaning is that many of them might not have known about the existence of dental floss and other interdental cleaning devices. This finding implies that the importance of cleaning teeth after meals, interdental cleaning as well as the use of appropriate interdental cleaning agents should be a major part of health education activities in school oral health programs. In addition, advocacy can be extended to companies manufacturing toothbrushes to include interdental cleaning devices as part of the tooth cleaning kit that would be sold to adolescents at subsidized rates.

The female gender was associated with more frequent tooth cleaning in this study. This has been reported by others (28–30). Females are known to pay more attention to their health including oral health and have better oral health (32). Notably, male students utilized dental care services more than females. This could be because of problems associated with inadequate care as dental care-seeking behavior is problem-driven in this environment and other regions (33, 34). Perhaps, it could be a feature of good oral hygiene habits exhibited by male adolescents in this study, moreover, male adolescents have been observed to have better oral health knowledge and attitude in the same city (16) and utilized dental services more than females in a suburban region in Nigeria (25). Adolescents whose parents were of a high occupational class cleaned their teeth more frequently than others. The influence of socioeconomic class on oral hygiene practices has been reported (28, 30, 35, 36). The importance of social class should be taken into cognizance when conducting oral health promotion programs in schools. Furthermore, adequate support in terms of the provision of tooth cleaning materials and encouraging the development of effective and cheaper alternatives appropriate for adolescents from low social classes could be helpful in minimizing existing inequality.

The impact of oral conditions on the QoL was high with over 90% reporting at least an impact on OHRQoL. This is higher than the impact of OHRQoL previously reported among adolescents in this environment: 21.1% among those aged 9–12 years (37), 21.4% among ages 6–15 years (38), 41.4% in those aged 10–13 years (39), and 51.5% among ages 13–15 years (40). Lower values of 57.4%–67.9% were also reported among 16–19-year-old Albanian adolescents (41) and 57.8%–60.8% in 10–11-year-old Malaysian adolescents (42). The varying distribution of oral diseases as well as the age of the study population may be responsible for the differences observed. Older adolescents are more conscious of their health compared to the younger age group (26), leading to a higher likelihood of reporting negative effects of health conditions on them. This finding may also be explained in part by toothache being the most frequently reported item of the COHIP-SF 19 measure and could explain the enthusiasm of the adolescents based on the high response rate for the study. The results showed that adolescents who cleaned their teeth more frequently were more likely to report no impacts on their quality of life than those who cleaned their teeth less frequently. Frequent teeth cleaning is a positive oral hygiene habit, which clears the mouth of dental plaque, the primary causative factor for periodontal diseases and dental caries (43). On the contrary, engaging in interdental cleaning was associated with reporting impacts on the OHRQoL. This may be associated with the presence of periodontal pocketing and food packing interdentally, driving the adolescents to engage in cleaning the interdental areas of the teeth. In addition, it may be attributed to the use of harmful objects such as knives and broomsticks that result in the inflammation of the gingiva and subsequent oral diseases from such habits. The inclusion of interdental cleaning as a key component of oral health education programs among adolescents thus, becomes pertinent. The period and duration of tooth cleaning were not associated with OHRQoL. Although spending longer time and cleaning after meals are good oral hygiene habits that result in plaque-free mouth, the finding of the study as regards these two variables not associated with OHRQoL could be related to the quality of tooth cleaning. Correct tooth cleaning method has been implicated as an important factor in plaque removal from the mouth (43), thus preventing oral diseases that are known to impact the quality of life.

## Conclusion

The oral hygiene habits of the adolescents were suboptimal and those who cleaned their teeth more frequently had fewer impacts on their OHRQoL, whereas those who engaged in interdental cleaning had higher impacts on their OHRQoL than others. Further studies to validate the findings of this study and the use of qualitative methods to explore the perspectives of the adolescents about oral hygiene habits and OHRQoL would assist in a better understanding of how these factors affect their OHRQoL.

## Data Availability

The raw data supporting the conclusions of this article will be made available by the authors, without undue reservation.
