# Supplementary material for: Impact of oral hygiene habits on oral health-related quality of life of in-school adolescents in Ibadan, Nigeria
Source: Front Oral Health. 2022 Sep 9;3:979674. doi: 10.3389/froh.2022.979674 (PMC9632948; doi:10.3389/froh.2022.979674)
Supplement: Supplementary file 1 [file Datasheet1.pdf]

Questionnaire

Dear student,

This questionnaire is designed to assess your oral hygiene habits and oral health related quality of life. Your honest answers will be treated with upmost confidentiality. Thank you.

Sociodemographic characteristics. Please fill / tick as appropriate

1. Age as at last birthday ---years
2. Gender 1) Male 2) Female
3. Father's Occupation---
4. Mother's occupation---

Oral hygiene habits: Please tick as appropriate.

5. How many times do you clean your teeth? 1) None 2) once 3) twice or more 4) not everyday
6. How many times do you clean your teeth? 1) None 2) once before breakfast 3) twice before breakfast and dinner 4) after each meal 5) after breakfast and dinner 6) before breakfast and after dinner 7) others --------------------------------
7. What do you use mainly to clean your teeth? 1) chewing stick 2) toothbrush 3) cotton wool 4) nothing 5) others --------------------------------
8. What kind of toothbrush do you use? 1) Soft 2) medium 3) hard 4) very hard 5) don't know
9. How many minutes to you spend cleaning your teeth? 1) < 1 minute 2) 1 minute 3)2 minutes 4) 3 minutes or more 4) don't know
10. Do you clean between your teeth? 1) Yes 2) No
11. What do use to clean between your teeth? 1) toothpicks 2) broom sticks 3) blade 4) dental floss 5) interdental brush 6) others please specify -------------------------
12. When do you change your toothbrush? 1) less than 3months 2) 3months 3) more than 3months 4) when bristles are fraying 5) one year or more than one year
13. Have you been to the dentist before? 1) Yes 2) No

Oral-health-related-quality-of-life ; Please tick as appropriate

14. How would you rate the present condition of your mouth and teeth?

a) Very bad b) bad c) I don’t know d) Good e) Very good

In the last three months: How often have you

	Almost all of the time	Fairly often	Sometimes	Almost never	Never”
Had pain in your teeth/toothache					
Had discoloured teeth or spots on your teeth					
Had crooked teeth or spaces between your teeth					
Had bad breath					
Had bleeding gums					
Had difficulty eating foods you would like to eat					
Had trouble sleeping					
Had difficultly saying certain words					
Had difficulty keeping your teeth clean					
Been unhappy or sad					
Felt worried or anxious					
Avoided smiling or laughing with other children					
Felt that you look different					
Been worried about what other people think about your teeth/mouth					
Been teased, bullied, or called names by other children because of your teeth					
Missed school for any reason because of your teeth/mouth					
Not wanted to speak/read out loud in class because of your teeth /mouth					
Been confident					
Felt that you were attractive (good looking)					
